# Association between hypertensive patients of Kazakh nationality in Xinjiang and T lymphocytes microRNAs

**DOI:** 10.1097/MD.0000000000046063

**Published:** 2026-05-12

**Authors:** Jian Dong, Min Jiao, Yajing Chao, Bin Gao, Yuanming Zhang

**Affiliations:** a Department of Cardiology, The First Affiliated Hospital of Xinjiang Medical University, Urumqi, Xinjiang Uygur Autonomous Region, China; b Department of Pharmacy, People’s Hospital of Xinjiang Uygur Autonomous Region, Urumqi, China; c Department of Rheumatology and Immunology, Xi’an Daxing Hospital, Yan’an University, Xi’an, Shaanxi, China; d Heart Center, The 7’th People’s Hospital of Zhengzhou, Zhengzhou, Henan, China.

**Keywords:** essential hypertension, miRNA, T lymphocyte

## Abstract

To explore the relationship between microRNAs (miRNAs) in T lymphocytes and primary hypertension in Kazakh people. Five Kazakh patients with primary hypertension who were initially diagnosed and untreated, as well as 5 healthy individuals who underwent physical examinations during the same period, were selected. Plasma samples of the subjects were collected, and T lymphocytes were isolated. High throughput second-generation sequencing technology was used to detect changes in miRNA expression in the two groups of subjects, and differentially expressed miRNAs were screened for further analysis of lymphocytes IL-6, IL-17, and interferon-gamma (IFN-γ), tumor necrosis factor-alpha (TNF-α) mRNA expression level detection; Serum IL-6, IL-17, IFN-γ, TNF-α detection of cytokine levels; Correlation analysis and clinical value prediction between differential expression of miRNAs and mean arterial pressure in patients. A total of 1754 miRNAs were detected in this study. Compared with the control group, 33 miRNAs were upregulated in the hypertension group (*P* < .05), and 60 miRNAs were downregulated (*P* < .05). Verify 6 miRNAs in the sample population: miR-15b-5p, miR-374-5p, miR-138-5p, miR-320a-3p, miR-423-5p, and miR-92a-3p. The quantitative real-time polymerase chain reaction results showed that among the 6 differentially expressed miRNAs, miR-320a-3p, miR-423-5p, and miR-92a-3p were downregulated in the hypertensive group (*P* < .05). Lymphocyte IFN in hypertensive group-γ, The expression level of IL-17 mRNA increases, and IL-6 and IFN in plasma-γ, The level of IL-17 has increased. The area under the miRNAs curve for differential expression of lymphocytes in Kazakh hypertensive patients, where miR-423-5p, area under the curve (AUC): 0.696 (95% confidence interval (CI): 0.533–0.859); MiR-320a-3p, AUC: 0.764 (95% CI: 0.609–0.919). The expression of miR-92a-3p, miR-423-5p, and miR-320a-3p in T lymphocytes of Kazakh hypertensive patients is downregulated, with good diagnostic reference value (*P* < .05).

## 1. Introduction

Essential hypertension as a common cardiovascular disease, there are many causes of hypertension. Since the role of lymphocytes in the development of hypertension was determined, researchers have been continuously exploring the functional role of lymphocytes in the pathogenesis of hypertension.^[1]^ Usually, in the body, the aggregation and activation of lymphocytes caused by various diseases or exogenous stimuli damage or repair the body by promoting the release of corresponding cytokines.^[2]^ The effector molecule of the renin angiotensin system, angiotensin II, induces hypertension. It not only regulates vascular tension and sodium balance, but also activates immune cells, promoting cell infiltration into target organs.^[3]^ microRNAs (miRNAs) have been found to be new signaling molecules that regulate gene expression after transcription. The complex network regulation relationship between miRNAs and target genes is involved in various important cell functions and disease processes. New research evidence also suggests that miRNAs are a novel class of inflammatory regulatory factors that regulate pro-inflammatory effects at different levels of cascade pro-inflammatory signaling. miRNAs also affect the differentiation and function of T cells. It has been found that miR-15a/16-1, miR-125b-5p, miR-99a-5p, miR-128-3p, let-7 family, miR-210, miR-182-5p, miR-181, miR-155, and miR-10a play a role in disease development by activating different types of lymphocytes to release corresponding pro-inflammatory factors, or regulating cell activity and inducing apoptosis.

Inflammatory mediators known as cytokines are released by immune cells, endothelial cells, and epithelial cells after injury or stimulation. Different immune cells have different functions during hypertension or terminal organ damage. Currently, humans have characterized over 120 immune cell subsets. After stimulation such as angiotensin II or a high salt diet that triggers a hypertensive response, the innate immune system contacts and activates the cells of the adaptive immune system.^[3]^ For example, in the subset of T cells, CD8^+^ T cells express IFN-γ, tumor necrosis factor-alpha (TNF-α), and IL-12.^[4]^ In CD4^+^ T helper cells, pro-inflammatory Th1 cells produce IFN-γ And TNF-α^[5]^; Th2 cells release IL-4, IL-5, and IL-13^[6]^; Th17 produces prohypertensive cytokines IL-17 and IFN-γ^[7]^ and regulatory T cells secrete immunosuppressive cytokines IL-10 and transforming growth factor-beta.^[8]^

The above previous research results have fully demonstrated the important role of lymphocytes in the formation of hypertension, and miRNAs in lymphocytes also have a certain pathogenic effect. In our previous research, we found that lymphocytes in hypertensive patients of the Kazakh ethnic group in Xinjiang were activated and accompanied by IL-6 and TNF-α. As the level of inflammatory factors increases, the activation of lymphocytes may be related to the activation of potassium ion channels. The regulation of lymphocyte activation by miRNAs has been confirmed. The functional role of miRNAs derived from lymphocytes in hypertension has not been documented. On the basis of previous research, this study further investigates the relationship between lymphocyte miRNAs and hypertension in Kazakh hypertensive patients.

## 2. Methods

### 2.1. Human participants and serum samples

From June 2020 to March 2021, in the First Affiliated Hospital of Xinjiang Medical University, Kazakh hypertensive patients who were newly diagnosed with hypertension or had previously been diagnosed with hypertension but did not receive treatment, as well as Kazakh healthy individuals with normal blood pressure during the same period of time, underwent physical examinations. This study divided the sample into two parts. In the first part, 5 Kazakh hypertensive patients and 5 healthy individuals were selected to form a sequencing set. The validation set consists of 40 Kazakh hypertensive patients and 40 healthy examinees in the second part. The included patients were diagnosed with hypertension for the first time according to the 2018 Chinese Guidelines for the Diagnosis and Treatment of Arterial Hypertension (i.e., resting systolic blood pressure ≥ 140 mm Hg and/or diastolic blood pressure ≥ 90 mm Hg). Exclusion criteria include receiving hypertension related drug treatment (i.e., lipid-lowering drugs, antiplatelet or antihypertensive drugs), hyperlipidemia, diabetes, hyperhomocysteinemia, coronary heart disease, cerebral infarction, stroke, chronic obstructive pulmonary disease, liver and kidney failure, severe infection, and malignant tumor.

All research subjects have signed informed consent forms, and all operating procedures in this study have been approved by the Ethics Committee of the First Affiliated Hospital of Xinjiang Medical University, meeting the ethical review requirements.

### 2.2. Experimental grouping

Control group: systolic blood pressure ≤ 120 mm Hg, diastolic blood pressure ≤ 80 mm Hg; Hypertension group: systolic blood pressure > 140 mm Hg, diastolic blood pressure > 90 mm Hg.

### 2.3. Isolation of T lymphocyte

Peripheral blood mononuclear cells were isolated using Ficoll–Hapaque density gradient centrifugation, and cultured in 1640 medium containing 10% Fetal Bovine Serum and were incubated at 37℃ in a 5% CO_2_ incubator. T lymphocytes were isolated using an immunomagnetic bead–based positive selection method (Miltenyi Biotec, Germany). Briefly, peripheral blood mononuclear cells were resuspended in phosphate-buffered saline (PBS) containing 0.5% Bovine Serum Albumin and 2 mM EDTA, and incubated with anti-CD3 monoclonal antibody–conjugated magnetic beads (20 μL beads per 1 × 10^7^ cells) at 4°C for 15 minutes. The cell suspension was then applied to a magnetic separation column according to the manufacturer’s instructions. After washing 3 times with buffer, CD3^+^ T cells were eluted and collected. The purity of isolated T lymphocytes was confirmed to be >90% by flow cytometry. Although immunomagnetic bead separation provides high purity and reproducibility, it may partially activate T lymphocytes during incubation, and the yield and purity depend on sample quality and operator technique. In addition, this method isolates total T lymphocytes but does not distinguish between CD4^+^ and CD8^+^ subsets, which could influence subsequent functional analyses.

### 2.4. Illumina sequencing method

#### 2.4.1. Extraction of total RNA from cells

The cells were collected into a 15 mL centrifuge tube and centrifuged at 1000 times, then the supernatant was discarded. After counting the cells, wash the cells with PBS precooled in advance, centrifuge at 1000 rpm and discard the supernatant; resuscitate the cells with aseptic PBS buffer precooled at 4℃, transfer to 1.5 mL enzyme-free Eppendorf, centrifuge for 5 minutes at 1000 rpm, discard the supernatant; add 0.5 to 1 mL Trizol lysate to the Eppendorf tube, fully blow, and stand at room temperature for 10 minutes; add 200 µL chloroform, severe concussion for 1 minute, static at room temperature for 10 minutes, 4℃, 12,000 rpm centrifugation for 15 minutes. Carefully absorb the upper water phase to the new tube, add the same amount of isopropanol and mix well, leave it in the refrigerator at −20℃ for 5 to 10 minutes, centrifuge at 4℃ and 12,000 rpm for 15 minutes; discard the supernatant and add 75% ethanol 1 mL to wash the ribonucleic acid (RNA) precipitation. Centrifuge 10 minutes at 4℃ for 7500, discard the supernatant, dry the air, and add 10 to 20 μL diethylpyrocarbonate water to dissolve the RNA precipitation.

#### 2.4.2. Detection of total RNA purity of T lymphocytes

0.3 g agarose reagent was added to the 1× Tris-Borate-EDTA electrophoresis buffer of 20 mL to configure agarose gel, and 1 mL EB dye was added as an indicator. 1 mL 10× sample buffer was fully mixed with 5 mL total RNA for gel electrophoresis. The electrophoretic condition was 10 minutes at 5 V pinch cm. After electrophoresis, the results were scanned and recorded by ultraviolet gel imager.

### 2.5. RT-qPCR

For quantitative real-time PCR, total RNA and miRNA were extracted by using TRIzol reagent (Invitrogen). For serum samples, RNA was isolated by using TRIzol from 200 µL serum. Te messenger ribonucleic acid (mRNA) or miR expression was determined by using SYBR Green (Bio-Rad) or TaqMan probe-participated quantitative real-time polymerase chain reaction (qPCR); β-actin and U6 were internal controls for normalization of mRNA and miR expression, respectively. Te sequences for qPCR primers are in Table 1.

**Table 1:** Probe, primer, and product (bp) for qPCR.

Gene	Upstream primer/Downstream primer
has-miR92a-3P	F: UAUUGCACUUGUCCCGGCCUGU
has-miR374a-5P	F: UUAUAAUACAACCUGAUAAGUG
has-miR320a-3p	F: AAAAGCUGGGUUGAGAGGGCGA
has-miR423-5P	F: UGAGGGGCAGAGAGCGAGACUUU
has-miR15b-5P	F: UAGCAGCACAUCAUGGUUUACA
has-miR-138-5P	F: AGCUGGUGUUGUGAAUCAGGCCG
U6	F: CTCGCTTCGGCAGCACA
IL-17A	F: GAGGACAAGAACTTCCCCCG
	R: CTTGCTGGATGGGGACAGAG
IL-6	F: TTTCAGGGTTGTGGAATCTT
	R: AGTGGGGGTCTTGCCAGGTG
TNF-α	F: GTGCTCCTCACCCACACCATA
	R: AAGACCCCTCCCAGATAGATA
IFN-γ	F: GAGATGACTTCGAAAAGCTGAC
	R: CCTTTTTCGCTTCCCTGTTTTA
ACTIN	F: TGGCACCCAGCACAATGAA
	R: CTAAGTCATAGTCCGCCTAGAAGCA

IFN-γ = interferon-gamma, qPCR = quantitative real-time polymerase chain reaction, TNF-α = tumor necrosis factor-alpha.

### 2.6. Determination of serum amylase, IL-6, IL-17, IFN-γ and TNF-α levels

Serum, IL-6, IL-17, interferon-gamma (IFN-γ) and TNF-α levels were measured using enzyme-linked immunosorbent assay kits (R&D Systems, Minneapolis).

### 2.7. Quality control

(1) The subjects included in this experiment were screened strictly according to the inclusion/ exclusion criteria.
(2) In this study, the original sequencing data will first remove the 3 ‘junction sequence and remove the sequence whose base length is less than 18 nt. If there are 80% An or C or G or T 3N (not necessarily continuous) in the sequence, only An and C do not have G, T, or only G and T do not have A, C or continuous nucleotide dimer, trimer, these sequences will be filtered out.
(3) Annotate the small RNA obtained by sequencing with RFam (including rRNA, tRNA, snRNA, snoRNA, etc), and remove the possible rRNA, snoRNA, snRNA, tRNA and other non-miRNA sequences as much as possible. Then select the small RNA obtained from the Repbase database annotation sequencing, compare and filter, and go out repeat associates RNA as much as possible. The filtered data were further identified and predicted by miRNA comparison.
(4) Based on the analysis and statistics of the original sequencing data, the length distribution statistics of the effective data after screening should be carried out. If most of the data are distributed in 20 to 24 nt, it accords with the typical characteristics of Dicer cleavage.

### 2.8. Statistical analysis

Analyses were performed with GraphPad Prism 9. For normally distributed data, the two-tailed Student *t* test was used to compare two groups and analysis of variance with Bonferroni post hoc test for multiple groups. For non-normally distributed data, the Mann–Whitney *U* test was used to compare two groups and Kruskal–Wallis test for multiple groups. Correlational analyses involved using Spearman correlation. Data are expressed as mean ± standard error of mean and *P < .05 was considered statistically significant.

Missing data were minimal (<5%) and were handled by case-wise deletion to ensure data integrity. Sensitivity analyses suggested that the exclusion of cases with missing values did not materially change the results. Potential sources of bias include the relatively small sample size, single-center recruitment, and the lack of multi-ethnic comparison, which may limit the generalizability of the findings. However, strict inclusion and exclusion criteria and standardized laboratory protocols were applied to minimize selection bias and measurement bias.

## 3. Results

**Figure 1.**
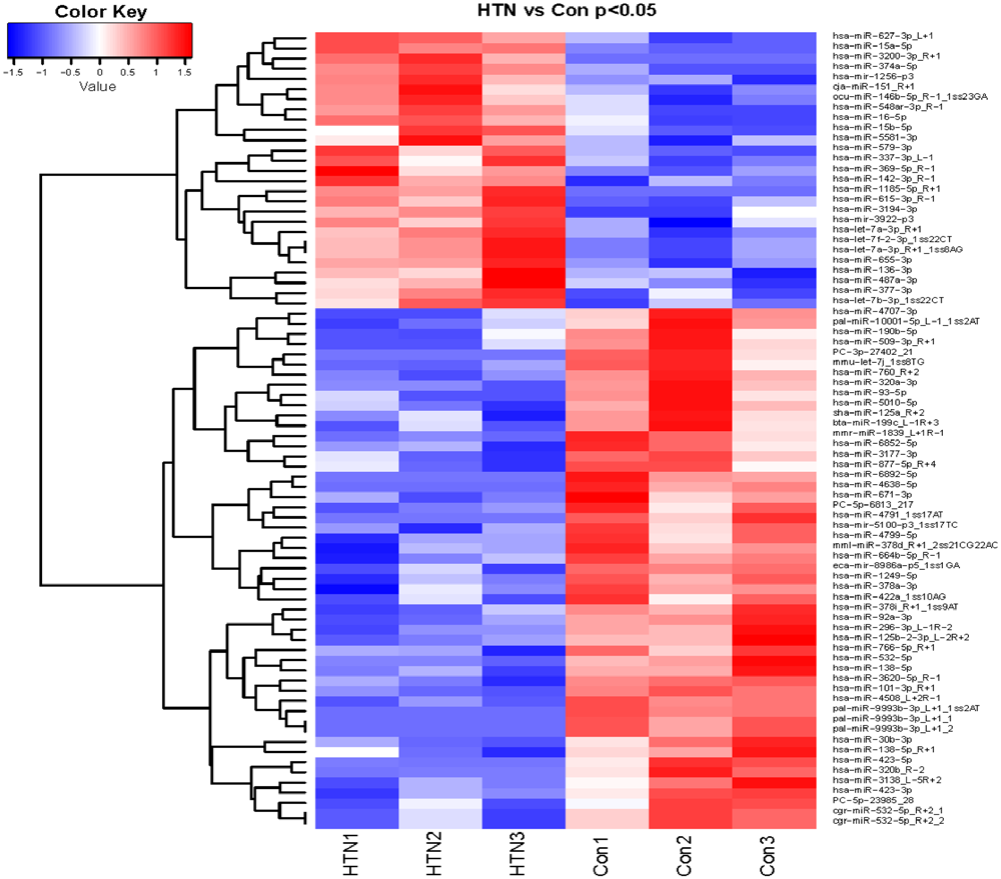
Heat map of clustering analysis of differentially expressed miRNAs. The horizontal axis is Log2 (expression value + 1) of different groups, and the vertical axis is miRNA. blue indicates down-regulated miRNA, red indicates up-regulated miRNA, darker blue indicates lower expression, darker red indicates higher expression. HTN is in the hypertension group and Con is in the healthy control group. miRNA = microRNA.

Selected subject characteristics are presented in Table 2. Other than high-density lipoprotein cholesterol, left ventricular end-diastolic diameter, interventricular septal thickness, there were no significant differences between groups in anthropometric or metabolic variables. The sequencing results showed that a total of 1754 miRNAs were detected, among which the number of downregulated miRNAs was significantly higher than that of upregulated miRNAs in the hypertension group. There were 27 upregulated miRNAs and 51 downregulated miRNAs with statistical differences (*P* < .05). Among them, 6 upregulated miRNAs and 9 downregulated miRNAs showed significant statistical differences (*P* < .01) (Fig. 1).

**Table 2:** General situation of the 4 groups of subjects ($\bar{x}\pm \;s$, n = 40).

Variable	Sequenced		*P*	Validation		*P*
	Normotensive (n = 5)	Hypertensive (n = 5)		Normotensive (n = 40)	Hypertensive (n = 40)	
Age (yr)	51.3 ± 6.4	51 ± 8.5	.96	48.5 ± 10.1	51.9 ± 8.9	.38
Sex (M/F)	3/ (60.0%)	3/ (60.0%)	1	18/ (45%)	30/ (75%)	.05
BMI (kg·m^−1^)	28.7 ± 2.4	33.7 ± 5.7	.23	26.0 ± 3.9	29.78 ± 3.9	.93
Systolic blood pressure (mm Hg)	113.7 ± 3.5	153 ± 3	.00	105.1 ± 6.8	163.6 ± 11.0	.02
Diastolic blood pressure (mm Hg)	69.3 ± 8.1	96 ± 9.6	.02	59 ± 12.6	102 ± 8.8	.04
FPG (mmol·L^−1^)	4.6 ± 0.7	4.4 ± 0.4	.75	4.7 ± 0.4	4.7 ± 0.7	.06
TG (mmol·L ^−1^)	1.7 ± 0.7	1.3 ± 0.4	.17	1.2 ± 0.6	2.1 ± 2.0	.08
LDL-C (mmol·L^−1^)	2.92 ± 0.6	2.57 ± 0.49	.47	2.77 ± 0.7	3.06 ± 0.8	.45
HDL-C (mmol·L^−1^)	0.97 ± 0.23	0.86 ± 0.19	.57	1.25 ± 0.3	1.05 ± 0.1	.02
Scr (mmol·L^−1^)	64.3 ± 5.5	81.5 ± 8.5	.04	74.96 ± 13.5	78.90 ± 15.8	.60
LA (mm)	32 ± 2.6	49.03 ± 9.84	.36	34.3 ± 3.3	35.5 ± 3.3	.73
LVEDD (mm)	46 ± 4	48.7 ± 3.1	.41	46.15 ± 4.17	49.7 ± 2.25	.01
IVST (mm)	9 ± 0	9.3 ± 0.6	.37	8.95 ± 0.39	9.4 ± 0.82	.00
EF (%)	63.8 ± 1.6	63.1 ± 1.1	.57	63.67 ± 2.43	61.59 ± 2.41	.90

BMI = body mass index, EF = ejection fraction, FPG = fasting plasma glucose, HDL-C = high-density lipoprotein cholesterol, IVST = interventricular septal thickness, LA = left atrium/left atrial, LDL-C = low-density lipoprotein cholesterol, LVEDD = left ventricular end-diastolic diameter, Scr = serum creatinine, TG = triglycerides.

### 3.1. Expression analysis of miR-15b-5p, miR-374-5p, miR-138-5p, miR-320a-3p, miR-423-5p, and miR-92a-3p on T lymphocytes in Xinjiang Kazakh hypertensive patients and control group

**Figure 2.**
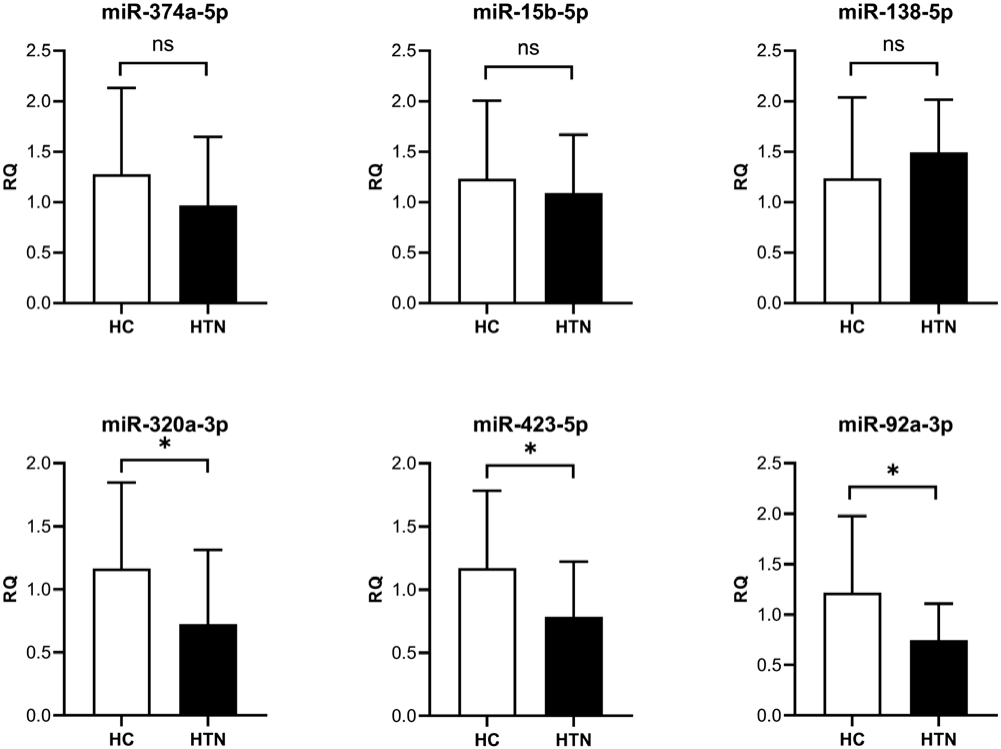
Differential expression of miRNAs in T lymphocytes from Kazakh hypertensive patients “*” *P* < .05. miRNA = microRNA.

Based on the multiple differences and literature, we selected 6 miRNAs: miR-15b-5p, miR-374-5p, miR-138-5p, miR-320a-3p, miR-423-5p, and miR-92a-3p for further validation in T lymphocytes of Kazakh hypertensive patients. The results showed that miR-320a-3p, miR-423-5p, and miR-92a-3p expressed miRNAs in lymphocytes of Kazakh hypertensive patients (Fig. 2, Table 3).

**Table 3:** RT-qPCR results of 6 differentially expressed miRNAs.

Variable	miR-15b-5p	miR-92a-3p	miR-138-5p	miR-374a-5p	miR-423-5p	miR-320a-3p
Normotensive	1.23 ± 0.77	1.22 ± 0.76	1.23 ± 0.80	1.28 ± 0.86	1.17 ± 0.61	1.17 ± 0.68
Hypertensive	1.09 ± 0.58	0.74 ± 0.36*	1.49 ± 0.52	0.97 ± 0.68	0.79 ± 0.43*	0.72 ± 0.59*

*Statistically significant difference. miRNA/ miR = microRNA, RT qPCR = quantitative real-time polymerase chain reaction.

### 3.2. Lymphocyte IL-6, IL-17, and IFN-γ, TNF-α in hypertensive Kazakh patients in Xinjiang miRNA expression level detection

**Figure 3.**
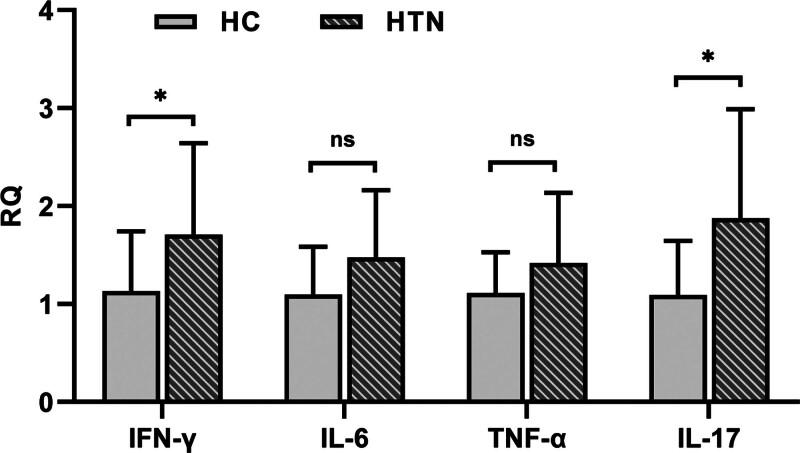
Expression levels of mRNA of inflammatory factors in T lymphocytes of Kazakh patients with hypertensions “*” *P* < .05. mRNA = messenger ribonucleic acid.

Perform RT qPCR detection on the expression of T lymphocyte specific inflammatory cytokine genes. Compared with the control group IL-17 (1.09 ± 0.55); IFN-γ compared with (1.13 ± 0.61), IL-17 (1.88 ± 1.11) and IFN in lymphocytes of Kazakh hypertensive patients-γ the gene expression level increased by (1.71 ± 0.93), and the difference was statistically significant (*P* < .05). Compared with the control group TNF-α (1.11 ± 0.42); Compared with IL-6 (1.1 ± 0.49), TNF in lymphocytes of hypertensive patients-α (1.42 ± 0.72); The expression of IL-6 (1.48 ± 0.68) gene showed an increasing trend in the hypertensive patient group, with no statistical significance (*P* > .05) (Fig. 3).

### 3.3. Serum IL-6, IL-17, IFN-γ, TNF-α cytokine level detection

**Figure 4.**
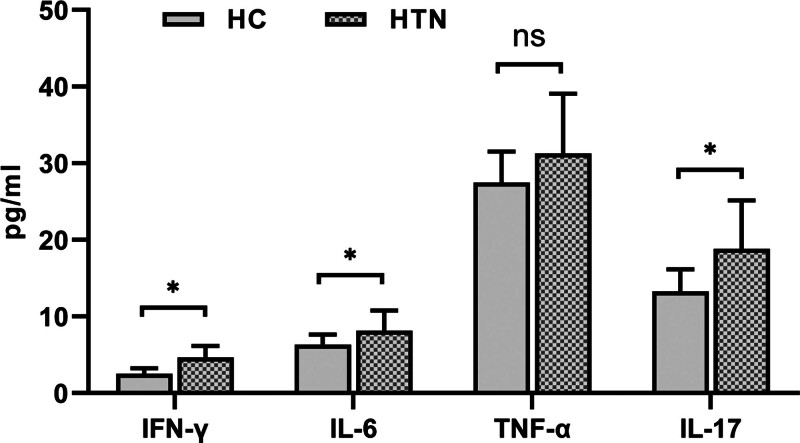
Serum inflammatory factor levels in Kazakh patients with hypertension “*” *P* < .05.

Detection of cytokine levels in the serum of Kazakh hypertensive patients, compared with the control group IL-6 (6.33 ± 1.31), IL-17 (13.28 ± 2.86), and INF-γ compared with (2.5 ± 0.74), IL-6 (8.14 ± 2.63), IL-17 (18.8 ± 6.33), and INF in the serum of hypertensive patients-γ (4.64 ± 1.48) the level of cytokines increased, and the difference was statistically significant (*P* < .05). Compared with the control group TNF-α compared to (27.5 ± 4.04), serum TNF in hypertensive patients-α there is an increasing trend (31.28 ± 7.76), but there is no statistical significance (*P* > .05) (Fig. 4).

### 3.4. Correlation analysis between differential expression of miRNAs and mean arterial pressure in Kazakh hypertensive patients in Xinjiang

**Figure 5.**
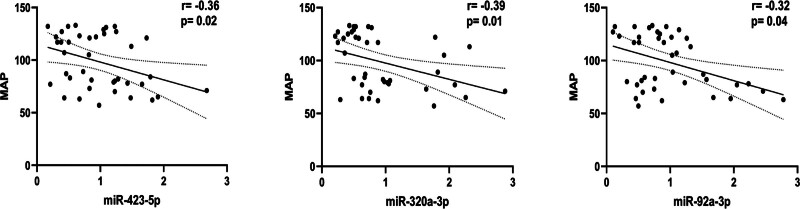
Correlation between differentially expressed miRNAs. miRNA = microRNA.

The elevated blood pressure levels in Kazakh hypertensive patients may be related to the downregulation of miR-92a-3p, miR-320a-3p, and miR-423-5plevels (Fig. 5).

### 3.5. Prediction of clinical diagnostic value of differentially expressed miRNAs in Kazakh hypertensive patients in Xinjiang

**Figure 6.**
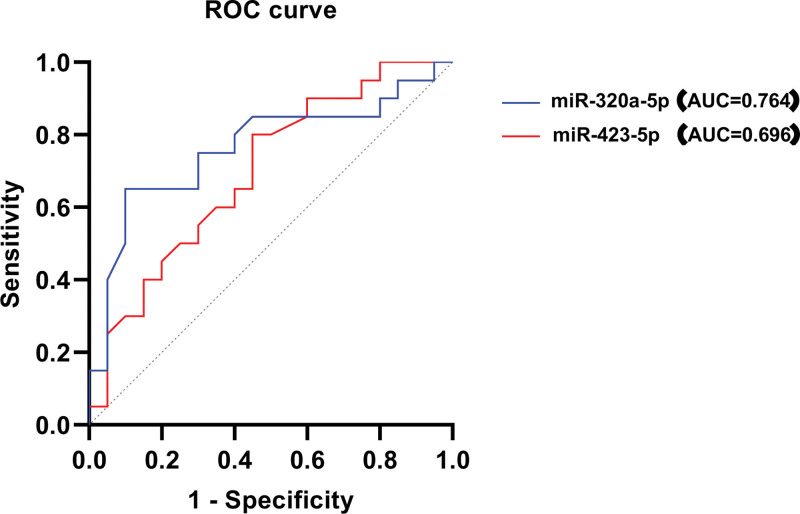
Subject characteristics curve subject curve.

Based on the sequencing results, 6 differentially expressed miRNAs were screened, and for hypertensive patients, miR-423-5p; The receiver operating characteristic (ROC) curves of miR-423-5p and miR-320a-3p showed area under the curve (AUC) values of 0.696 (95% confidence interval (CI): 0.533–0.859) and 0.764 (95% CI: 0.609–0.919), respectively. Although the 95% CI intervals are relatively wide due to the limited sample size, both miRNAs demonstrated moderate discriminatory ability in differentiating hypertensive patients from healthy controls. Clinically, an AUC of 0.70 to 0.80 is generally considered acceptable diagnostic performance, suggesting that these T lymphocyte–derived miRNAs may serve as potential auxiliary biomarkers for the early identification of hypertension in the Kazakh population. We further validated the discrimination and calibration of the model by simultaneously introducing miR-423-5p and miR-320a-3p into the logistic regression model: Model 1 is defined as miR-423-5p + miR-320a-3p; Model 2 is defined as miR-423-5p, with a Hosmer–Lemeshow test for calibration. Model 1 has a P1 value of 0.248 and Model 2 has a P2 value of 0.807, indicating that Model 2 has good calibration. The ROC curve is plotted using prediction probability for discrimination testing, with a C-index of 0.742 (95% CI: 0.586–0.899). The cut off value of miR-423-5p is calculated to be 1.15 (Fig. 6, Table 4).

**Table 4:** ROC analysis of differentially expressed miRNAs.

miRNAs	AUC	*P* value	95% CI	
			Lower bound	Upper bound
hsa-miR-92a-3p	0.66	.09	0.48	0.83
hsa-miR-423-5p	0.70	.03	0.53	0.86
hsa-miR-320a-3p	0.76	.00	0.61	0.92
hsa-miR-374a-5p	0.62	.21	0.44	0.79
hsa-miR-15b-5p	0.54	.69	0.35	0.72
hsa-miR-138-5p	0.35	.11	0.18	0.53

AUC = area under the curve, CI = confidence interval, miRNA/ miR = microRNA, ROC = receiver operating characteristic.

## 4. Discussion

miRNAs are important regulatory factors widely distributed in the human body. The relationship between miRNAs and hypertension is also very close.^[9,10]^ More and more evidence supports the important role of miRNAs in the pathogenesis of neointimal lesions, atherosclerosis, coronary artery disease and other hypertension associated diseases.^[11–14]^ Research has shown that increased expression of miRNAs in the plasma and extracellular vesicles of hypertensive patients can reduce inflammation.^[15,16]^ T lymphocytes have always been considered the most important pathogenic pathway of inflammation in the pathological development of hypertension. Previous studies have identified the roles of cytotoxic T cells (CD8^+^) and helper T cells (Th, CD4^+^) in hypertension.^[17,18]^ Th1, Th2, and Th17 cells can promote IFN-γ, the expression of inflammatory factors such as IL-17 and IL-22 leads to the occurrence of hypertension, and regulatory T cells can alleviate the occurrence of hypertension through IL-10 mediated functions.^[19]^

Hypertension is an important risk factor for a variety of cardiovascular and cerebrovascular diseases. Some studies have found that the prevalence of hypertension among Kazakh residents in Xinjiang is higher than that among Han residents in the same region, which may be related to the differences in dietary and living habits between residents of the two ethnic groups, but the specific biological mechanisms are still unclear. miRNA is a regulatory non-coding RNA, a class of single-stranded RNA molecules encoded by endogenous genes, with a length of about 19 to 23 nucleotides, which can be involved in the post-transcriptional level of gene expression regulation in animal and plant life activities. The present study found that the occurrence of hypertension is closely related to the regulation of miRNA. However, so far, the interaction between miRNAs and T lymphocytes in immune response as a new and important mechanism has not received attention.

Our previous research found that in Kazakh hypertensive patients in Xinjiang, the activation level of T lymphocytes is high, and some cytokines including TNF-α, IL-6 and other substances have higher levels in the plasma of Kazakh hypertensive patients, which is related to the activation of lymphocytes in Kazakh hypertensive patients. Therefore, this study takes Kazakh hypertensive patients as the research object, collects T lymphocytes from peripheral blood of Kazakh hypertensive patients and healthy control population, and further sequencing and analysis of the expression of miRNAs in T lymphocytes to screen for differentially expressed miRNAs. Then, further verification is carried out in Kazakh hypertensive population using bioinformatics methods, On the one hand, to explore the diagnostic value of miRNAs in lymphocytes of Kazakh hypertensive patients for primary hypertension, and on the other hand, to explore the potential role of target genes corresponding to differentially expressed miRNAs in the occurrence of hypertension.

T cell-derived cytokines play a central role in the pathophysiology of cardiovascular disease and hypertension, leading to terminal organ damage.^[20–22]^ One of the earliest discovered cytokines associated with hypertension is IL-17, which increases blood pressure and reduces NO dependent diastolic response by activating RhoA/Rho kinases.^[23]^ We found that the expression level of IL-17 mRNA in lymphocytes increased in Kazakh hypertensive patients, and serum IL-17 levels also increased compared to the control group. IL-6 can promote the polarization of CD4 T cells to produce IL-17 signaling, and exogenous increase in angiotensin II levels can promote an increase in IL-6 levels. Treatment with aldosterone antagonist spironolactone can block this process, which may be related to the activation of mineralocorticoid receptors.^[24]^ Previous studies have found that the activation of IL-6 by hypertensive lymphocytes tends to have a pathological effect on kidney injury. In our experimental results, the IL-6 mRNA levels in lymphocytes of hypertensive patients increased, although there was no significant difference, which may be due to the absence of typical kidney injury in the sample population of hypertensive patients. Other clearly defined adaptive immune cytokines in hypertension include IFN-γ and TNF-α in our study,^[25–27]^ IFN-γ MRNA levels in lymphocytes and IFN in serum-γ the levels are all upregulated in the hypertensive group, and activated lymphocytes promote the expression and release of inflammatory factors during the formation of hypertension, which may be directly related to terminal organ damage in hypertension. In summary, the results of this study show that lymphocytes IL-17 and IFN in Kazakh hypertensive patients-γ. Increased mRNA expression and inflammatory factors IL-17 and IFN in plasma-γ. The increase in IL-6 levels in hypertensive patients indicates that lymphocytes in Kazakh hypertensive patients are activated and produce pro-inflammatory effects.

Six differential miRNAs were identified in lymphocytes of Kazakh hypertensive patients through miRNA sequencing, including miR-15b-5p, miR-374-5p, miR-138-5p, miR-320a-3p, miR-423-5p, and miR-92a-3p. Compared with previous studies, some miRNAs have been reported to be involved in hypertension, including miR-92a-3p, miR-15b-5p, and miR-423-5p. In this study, there was a difference in the expression of miR-92a-3p on T lymphocytes between the hypertensive group and the control group, with miR-92a-3p expression downregulated in the hypertensive group. In previous hypertension related miRNA studies, the level of miR-92a in plasma was considered as a potential noninvasive biomarker of atherosclerosis in basic hypertension.^[28]^ In our study, lymphocyte miR-92a-3p was down regulated in hypertension group. Combined with the multiple roles of miR-92a-3p in the regulation of vascular function in previous studies, miR-92a-3p has a close relationship with vascular function damage and atherosclerosis in the process of hypertension formation. A study has detected high expression of miR-92a in the peripheral blood of patients with cardiovascular diseases. miR-92a is mainly expressed in the endothelial cells of blood vessels, and plays a pivotal role in the maintenance of vascular integrity and endothelial cell homeostasis, as well as in the control of angiogenesis, vascular damage, atherosclerosis, and plaque development.

MiR-92a-3p, miR-320a-3p, and miR-423-5p are downregulated in the Kazakh population with hypertension, indicating that it was related to the severity of the disease and could reflect the severity of vascular lesions to a certain extent. In addition, the formation of hypertension itself is multi-factorial, so it can also indicate that miRNAs is an important marker through different regulatory pathways to participate in and indirectly reflect the degree of hypertension and atherosclerosis progression. The ROC curve shows that miR-320a-3p and miR-423-5p have good predictive value for the onset of hypertension in the Kazakh population.

There are several limitations in this study. Firstly, the sample size for high-throughput sequencing was relatively small, which may have affected the robustness and statistical power of the results. Secondly, the study population was restricted to Kazakh individuals, and comparative analyses with other ethnic groups such as Han, Uyghur, or other populations in Xinjiang and other regions of China were not performed. As a result, the universality and generalizability of the findings are limited. In future studies, we plan to expand the sample size and include multiple ethnic groups to determine whether the observed alterations in T lymphocyte–related miRNAs are consistent across populations. Thirdly, although appropriate statistical methods were used, no correction for multiple comparisons (e.g., false discovery rate or Bonferroni adjustment) was applied at the initial screening stage, which might increase the risk of false positives given that 1754 miRNAs were detected. To mitigate this, we combined statistical thresholds with biological plausibility in candidate selection and further validated the most relevant miRNAs by RT-qPCR in an independent cohort, which strengthens the reliability of the key findings. Finally, certain methodological details, such as the potential limitations of the immunomagnetic bead–based T lymphocyte isolation procedure, may also influence reproducibility and should be interpreted with caution. Future studies with larger sample sizes, multi-center and multi-ethnic cohorts, and the application of stricter multiple testing corrections will be necessary to further validate and generalize these findings.

## 5. Conclusion

The expression of miR-92a-3p, miR-423-5p, and miR-320a-3p in T lymphocytes of Kazakh hypertensive patients is downregulated, leading to increased expression of inflammatory factors such as IL-17 and IFN-γ, the elevation of IL-6, and the occurrence of hypertension. These findings provide potential biomarkers for early diagnosis in this specific population. However, further multicenter studies including other ethnic groups are warranted to confirm whether these results can be generalized to broader populations.

## Author contributions

**Conceptualization**: Jian Dong, Min Jiao, Yajing Chao, Bin Gao, Yuanming Zhang.

**Data curation**: Min Jiao, Bin Gao, Yuanming Zhang.

**Formal analysis**: Min Jiao, Bin Gao, Yuanming Zhang.

**Funding acquisition**: Jian Dong, Yuanming Zhang.

**Investigation**: Jian Dong, Min Jiao.

**Methodology**: Jian Dong, Yajing Chao, Bin Gao.

**Supervision**: Min Jiao, Yajing Chao, Yuanming Zhang.

**Validation**: Min Jiao, Yajing Chao.

**Visualization**: Jian Dong, Min Jiao.

**Writing – original draft**: Jian Dong, Min Jiao, Yuanming Zhang.

**Writing – review & editing**: Jian Dong, Min Jiao, Yuanming Zhang.
